# Effects of Light and Wounding on Jasmonates in Rice *phyAphyC* Mutants

**DOI:** 10.3390/plants3010143

**Published:** 2014-03-03

**Authors:** Rita Brendel, Katharina Svyatyna, Yusuke Jikumaru, Michael Reichelt, Axel Mithöfer, Makoto Takano, Yuji Kamiya, Peter Nick, Michael Riemann

**Affiliations:** 1 Botanical Institute, Karlsruhe Institute of Technology, Karlsruhe 76131, Germany; E-Mails: rita.brendel@kit.edu (R.B.); katha.sv@gmx.de (K.S.); peter.nick@kit.edu (P.N.); 2 RIKEN Plant Science Center, Tsurumi, Yokohama, Kanagawa 230-0045, Japan; E-Mails: jikumaru@psc.riken.jp (Y.J.); ykamiya@postman.riken.jp (Y.K.); 3 Department of Biochemistry, Max Planck Institute for Chemical Ecology, Jena 07745, Germany; E-Mail: reichelt@ice.mpg.de; 4 Department of Bioorganic Chemistry, Max Planck Institute for Chemical Ecology, Jena 07745, Germany; E-Mail: amithoefer@ice.mpg.de; 5 Department of Plant Physiology, National Institute of Agrobiological Sciences, Tsukuba, Ibaraki 305-8602, Japan; E-Mail: mtakano@affrc.go.jp

**Keywords:** *Oryza sativa* L., jasmonate, jasmonate-isoleucine, JASMONATE RESISTANT 1, phytochrome, photomorphogenesis, mechanical wounding

## Abstract

Jasmonates (JA) are lipid-derived plant hormones. They have been shown to be important regulators of photomorphogenesis, a developmental program in plants, which is activated by light through different red and blue light sensitive photoreceptors. In rice, inhibition of coleoptile growth by light is a central event in photomorphogenesis. This growth inhibition is impaired, when jasmonate biosynthesis is knocked out. Previously, we found that *JASMONATE RESISTANT 1* (*OsJAR1*) transcripts were not induced in the phytochrome (phy) mutant *phyAphyC*. Therefore, in the current study we investigated the regulation of JA and its highly bioactive derivative (+)-7-*iso*-jasmonoyl-l-isoleucine (JA-Ile), as well as the transcriptional regulation of several JA-dependent genes both in wild type and *phyAphyC* mutant. JA and JA-Ile levels increased in the mutant seedlings in response to blue light. However, in *phyAphyC* mutant leaves, which were continuously wounded, JA and JA-Ile levels were lower compared to those in the wild type. Hence, the mutation of phyA and phyC has differential effects on jasmonate levels depending on the tissue and developmental stage. Our results suggest that the contribution of JA-Ile to signaling during photomorphogenesis of rice is minor, as coleoptile phenotypes of *phyAphyC* mutants resemble those of jasmonate-deficient mutants despite the fact that induction by blue light leads to higher levels of JA-Ile compared to the wild type. We postulate that phyA and phyC could control the activity of specific enzymes metabolizing JA to active derivatives.

## 1. Introduction

For plants it is crucial to respond to light fast as they use light as their main energy source. During the first days after germination they are able to sustain growth by consuming the nutrients stored in the seed. Thus, seedlings are able to grow rapidly in complete darkness although they are not photosynthetically active yet. During their passage through the soil, the leaves of rice seedlings are enclosed in an organ called coleoptile, which protects them from mechanical damage. Coleoptiles elongate very quickly in complete darkness, but are extremely light sensitive. Upon detection of light they stop growing and open along a predefined axis to release their leaves. This marks the transition of the seedling to a completely different developmental program, referred to as photomorphogenesis.

In order to perceive light, plants have evolved a sophisticated system of photoreceptors, which are either sensitive to blue (cryptochromes, cry; phototropin, phot [1,2,3,4]), or to red/far-red (phytochrome, phy; [5,6,7,8]) light. Phys exist in two forms, the red light absorbing P_r_- and the far-red light absorbing P_fr_-form, which both can be interconverted by irradiation of the appropriate wavelength. As rice harbors only three genes coding for the phys phyA, phyB, and phyC [9,10] it is convenient for functional studies of phys as compared to other model systems.

The three phytochromes differ in their kinetic properties after conversion into the P_fr_ form. While the P_fr_ form of phyB is stable in the light, phyA and phyC are degraded after activation, a phenomenon known as photodestruction [10]. For continuous irradiation, light qualities that establish a low photoequilibrium (the proportion of P_fr_ over total phytochrome), such as canopy light depleted in red light, are, therefore, more efficiently activating phyA/phyC signaling. In contrast for light qualities that establish a high photoequilibrium, such as daylight above the canopy, more P_fr_ is established than can be used for signaling and the excessive P_fr_ undergoes photodestruction and therefore is not contributing to sustain signaling [11]. However, very low fluence responses (e.g., germination), which are under control of phyA, also occur preferentially at a low photoequilibrium [12]. Due to these kinetic differences, continuous red light is activating almost exclusively phyB dependent signaling, whereas continuous far-red and blue light act through phyA/phyC.

The inhibition of coleoptile growth during photomorphogenesis is thought to be mediated by phys [13,14], but also an involvement of crys has been discussed recently [15]. Several examples show that the developmental changes during photomorphogenesis are regulated by plant hormones. For example in *Arabidopsis thaliana* brassinosteroids have been demonstrated to be important regulators of photomorphogenesis. When brassinosteroid biosynthesis is impaired, as in the *deetiolation 2 (det 2)* mutant, seedlings undergo photomorphogenesis in darkness [16,17]. In rice, jasmonates (jasmonic acid, JA, and derivatives) have been shown to be very important for the phy-dependent inhibition of coleoptile elongation [18,19,20]. Jasmonates are signaling compounds, which are biosynthesized from linolenic acid, cleaved from a chloroplast membrane lipid [21]. After conjugation to isoleucine (Ile) by JASMONATE RESISTANT 1 (JAR1) [22], JA is activated and can bind to its co-receptor CORONATINE INSENSITIVE 1 (COI1) in *Arabidopsis* [23,24]. Upon binding of COI1 and JA-Ile the complex can recruit JAZ proteins, repressors of jasmonate signaling [25,26]. Subsequently, JAZ proteins are presumably marked by ubiquitination for degradation in the proteasome. 

For rice, it has been shown that the deficiency of jasmonates leads to an altered photomorphogenesis. In the mutants *hebiba* and *coleoptile photomorphogenesis 2 (cpm2)*, the function of ALLENE OXIDE CYCLASE (AOC), a key enzyme of jasmonate biosynthesis, is impaired, leading to a long coleoptile phenotype in light [27]. Moreover, the photodestruction of phyA was found to be much slower in the *hebiba* mutant [28,29], which indicates a function for jasmonate in this process. 

JAR1 is important for the conversion of JA to the highly bioactive amino-acid conjugate JA-Ile and this enzymatic step has been shown to be light dependent in lima bean [30]. Therefore, mutants of the rice orthologue of JAR1 were studied. It was found that the long coleoptile phenotype of *osjar1* mutants was especially pronounced under blue light. However, this phenotype of *osjar1* mutants is less severe compared to the rice *aoc* mutants [31]. *OsJAR1* transcripts are inducible by blue light, but this induction is strongly reduced in *phyAphyC* mutants lacking the photodestructible isotypes of phytochrome [32]. 

The present study was motivated by this observation. We wanted to know whether the blue light induction of jasmonate biosynthesis and signaling is mediated entirely via phyA and phyC. Therefore, we investigated the regulation of the biosynthesis of JA and its derivative JA-Ile in *phyAphyC* mutants, as well as the regulation of JA-responsive genes. We compared the regulation of biosynthesis and signaling of jasmonate in response to light and mechanical wounding, a signal well-known to induce this plant hormone. In seedlings grown in blue light phyA and phyC might either attenuate the synthesis of JA or accelerate its metabolism, as, in mutants, both levels of JA and JA-Ile were elevated. In contrast, both compounds tended to be less abundant in wounded mutant compared to wild type leaves, indicating that the negative effect on JA and JA-Ile accumulation is specific for the seedling stage. 

## 2. Results and Discussion

### 2.1. The phyAphyC Mutant Displays a Phenotype in Response to Continuous Blue and Far-Red Light

**Figure 1:**
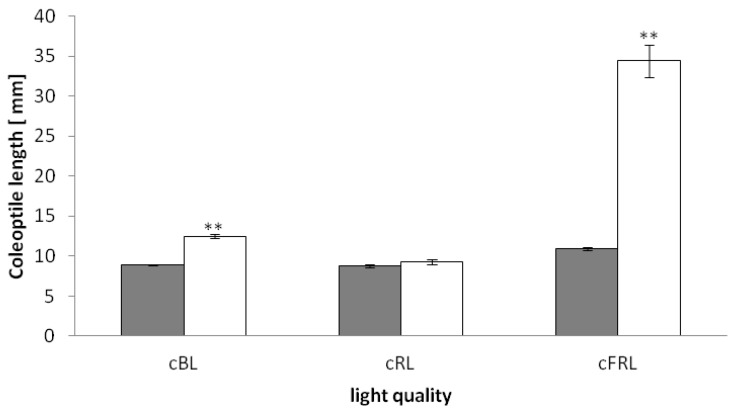
Length of rice coleoptiles after growth in continuous light of different qualities. cBL: continuous blue light (λ_max_ 470 nm), cRL: continuous red light (λ_max_ 650 nm), cFRL: continuous far red light (λ_max_ 735 nm), fluence rate was adjusted to 10 µmol/m^2^s for all light qualities. Coleoptile length of wild type (grey bar) and *phyAphyC* mutants (white bar) was measured after the leaves had pierced the coleoptile tip. Statistically significant differences to the wild type are indicated by two asterisks (Student’s *t*-test, *p* < 0.01).

In a recent study, where blue light was irradiated as a pulse of 3 min, it could be shown that the transcript for the JAR1-enzyme is not induced after blue light in etiolated *phyAphyC* mutant seedlings while its transcription is induced in the wild type [32]. Therefore, we compared the response of coleoptile growth of wild type and *phyAphyC* mutants to continuous irradiation with different light qualities. Wild type and *phyAphyC* mutant seedlings were grown in continuous blue (cBL), red (cRL), or far-red (cFRL) light and the coleoptile length were measured after the emergence of the leaves. Seedlings of *phyAphyC* mutants showed a longer coleoptile than the wild type in cFRL and cBL, while there was no such difference visible in cRL (Figure 1). 

As to be expected from the dependence of phytochrome photodestruction on light quality, the difference between wild type and *phyAphyC* mutant was strongest for far-red light. Under those conditions, mutant coleoptiles were in average about three times longer (34 mm *vs.* 11 mm) than those of the wild type. This result indicates a completely impaired de-etiolation under cFRL, which is in accordance with the findings of Takano *et al*. [10].

Interestingly, the *phyAphyC* mutants also showed a significant blue light phenotype. Grown in cBL, the coleoptile length was in average 12 mm compared to the wild type with only 9 mm. A similar, but stronger phenotype under blue light is also known from mutants, which are impaired in jasmonate biosynthesis, such as *hebiba*, *cmp2*, or *osjar1* [19,27,31]. In cBL, coleoptiles of *phyAphyC* mutants are shorter than those of *osjar1* mutants, which have the same genetic background, while the severely JA-deficient mutants *cpm2* and *hebiba* develop the longest coleoptiles [31].

### 2.2. The Blue-Light Response of OsJAR1 Is Specifically Suppressed in phyAphyC

As *phyAphyC* and the mutants deficient in JA or bioactive JA amino acid conjugates showed a similar blue light phenotype, we investigated the blue-light response of representative JA-responsive genes, known to be differentially expressed in *osjar1* mutants. 

The transcriptional regulation of genes for the biosynthesis of jasmonates (*OsAOC*, *OsJAR1*) and JA signaling (*OsTIFY10c/OsJAZ8*) [31,33] was analyzed in etiolated seedlings exposed to blue light for different incubation times (1 h, 2 h, and 6 h). *OsAOC* is a single-copy gene, while *OsJAR1* and *OsJAZ8* are members of gene families. *OsJAR1* belongs to the GH3 gene family consisting of 12 members in rice [34,35]. *OsJAZ8* belongs to the *TIFY* gene family consisting of 20 members in rice [36]. Out of those, 15 genes are putative *JAZ* genes.

**Figure 2:**
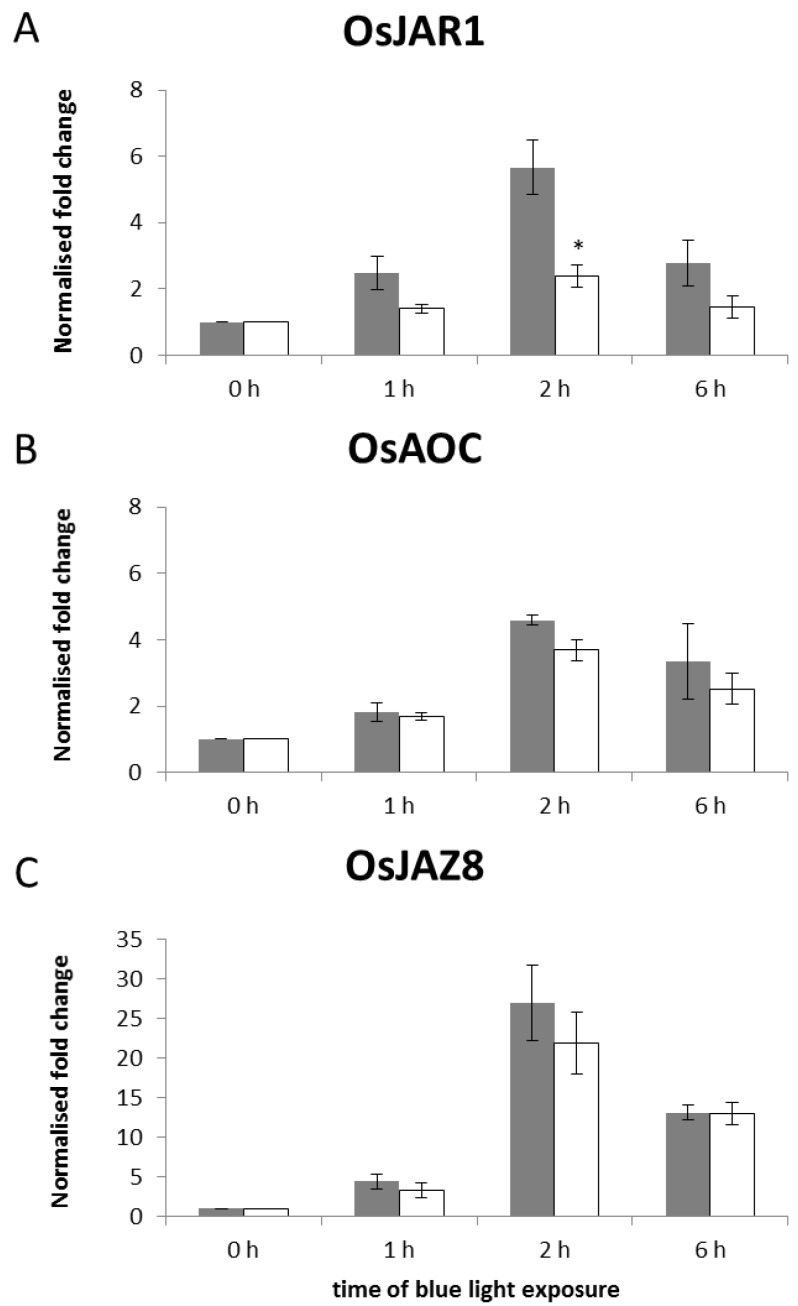
Transcript levels of the representative JA-biosynthesis genes *OsJAR1* (**A**) and *OsAOC* (**B**) and the JA-signaling gene OsJAZ8 (C) in wild type and mutant seedlings in response to blue light treatment. Nipponbare (grey bar) and *phyAphyC* mutant (white bar) seedlings were irradiated at day five with blue light (10 µmol/m^2^s) for the times indicated. qPCR analysis was carried out using two standard genes, eEF-1α and OsUBI5, for normalization. The fold change induction for each gene was calculated relative to the 0 h control plant, which was defaulted as 1. Data represent the average of three independent experiments. Statistically significant differences to the wild type are indicated by one asterisk (Student’s *t*-test, *p* < 0.05).

The transcript of *OsJAR1* is induced by blue light treatment in the wild type in a transient manner, peaking 2 h after the onset of irradiation relative to a dark control (six-fold induction in Nipponbare). Although this transcript is also induced in *phyAphyC*, the induction is much weaker (only two-fold, Figure 2A). This very low induction of *OsJAR1* in *phyAphyC* is in line with the results of Riemann *et al*. [32], who found, that *OsJAR1* transcript levels are basically not responsive to blue light in *phyAphyC* mutants. 

Similar to *OsJAR1*, the JA biosynthesis gene *OsAOC* is also induced transiently by blue light (Figure 2B), in both wild type and mutant *OsAOC* transcript levels are highest at two hours after a blue light pulse. 

In the JA-response gene *OsJAZ8* no significant difference between wild type and *phyAphyC* mutants was found (Figure 2C). This gene is induced strongly by blue light, also peaking at 2 h after irradiation (more than 20-fold) in both wild type and mutant. 

Thus, amongst the JA-responsive genes we have tested, exclusively the transcriptional regulation of *OsJAR1* was severely affected in *phyAphyC* mutants. It is likely that crys contribute to the transcriptional regulation of JA-dependent genes in blue light. Hirose *et al*. [15] have demonstrated that overexpression of *OsCRY1a* leads to an increased blue-light responsiveness of *OsALLENEOXIDESYNTHASE 1* transcription. The normal induction of *OsAOC* and *OsJAZ8* in the *phyAphyC* mutants might, therefore, be mediated via crys, since photolabile phytochrome is absent. A similar phenomenon has been reported for a mutant in *Arabidopsis* deficient in the chromophore of phytochrome, where, nevertheless, JA-induced genes remained responsive to light [37]. 

### 2.3. The phyAphyC Mutant Produces Higher Amounts of Jasmonates in Response to Blue Light

The transcription of *OsJAR1* was only weakly induced by blue light in *phyAphyC* (Figure 2A), and the coleoptiles of this mutant were longer in cBL (Figure 1). However, the two other JA-responsive genes basically retained their responsiveness to blue light in the mutant. Therefore, we examined the hormonal levels of JA and JA-Ile for selected time points during irradiation with blue light (dark, 1 h, 6 h). 

**Figure 3:**
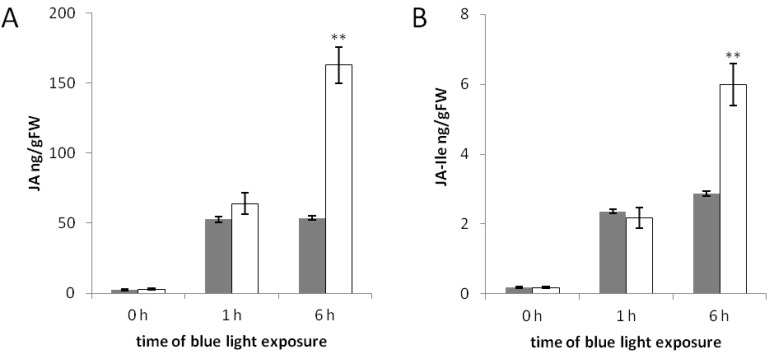
JA (**A**) and JA-Ile (**B**) levels of rice seedlings in response to blue light. Etiolated wild type (grey bars) and *phyAphyC* (white bars) seedlings were irradiated with blue light (10 µmol/m^2^s) at day five for the times indicated. Hormone levels are presented as the average of three independent samples relative to sample fresh weight. Statistically significant differences to the wild type are indicated by two asterisks (Student’s *t*-test, *p* < 0.01).

From the strongly reduced transcript levels for *OsJAR1* in the *phyAphyC* mutant, the abundance of JA-Ile is expected to be strongly reduced as compared to the wild type, whereas for JA a slight, but significant reduction was predicted. Surprisingly, in contrast to these predictions, *phyAphyC* produces higher levels of JA and JA-Ile after irradiation with blue light (Figure 3). One hour blue light irradiation induces the levels of JA and JA-Ile in both wild type and mutant to the same extent (Figure 3A,B). However, after six hours blue light, JA accumulates to almost three-fold higher (162.6 ng/gFW), and JA-Ile to two-fold higher (5.9 ng/gFW) levels in *phyAphyC* compared to the wild type (53.7 ng/gFW and 2.9 ng/gFW, respectively). An increased JA biosynthesis has also been reported for the already mentioned chromophore-deficient *Arabidopsis* mutant, while in the same study such an effect could not be found in a *phyAphyB* mutant [38].

Although JA and JA-Ile are overproduced in etiolated *phyAphyC* mutants subjected to blue light, these mutants nevertheless develop significantly longer coleoptiles in continuous blue light. On the other hand, mutants with impaired JA biosynthesis develop very long coleoptiles under blue light [31]. We conclude that a jasmonate derivative different from JA or JA-Ile controls coleoptile growth in response to blue light. This could be due to conjugation of JA to an amino acid different from Ile or an entirely different conjugate. In *Arabidopsis* it has been shown that methyl-JA (MeJA) is more effective in inhibiting hypocotyls elongation than JA-Ile [39], which suggests that JA derivatives different from JA-Ile promote growth inhibition in hypocotyls. In addition to JA, auxin may have a positive impact on coleoptile elongation of *phyAphyC* in blue light, as more auxin is present in the mutants (Figure A2). However, similar to the wild type, auxin levels in the mutants are decreasing. Therefore, the observed auxin effect may be a consequence of JA-dependent stimulation of auxin biosynthesis, which has been proposed previously [40]. It is surprising that, not only JA-Ile, but also JA is overproduced in *phyAphyC*, although the mutant develops longer coleoptiles. Hence, it might be possible that there is a phy-dependent, presently unknown step in the bioactivation of jasmonate in young rice seedlings. 

**Figure A1:**
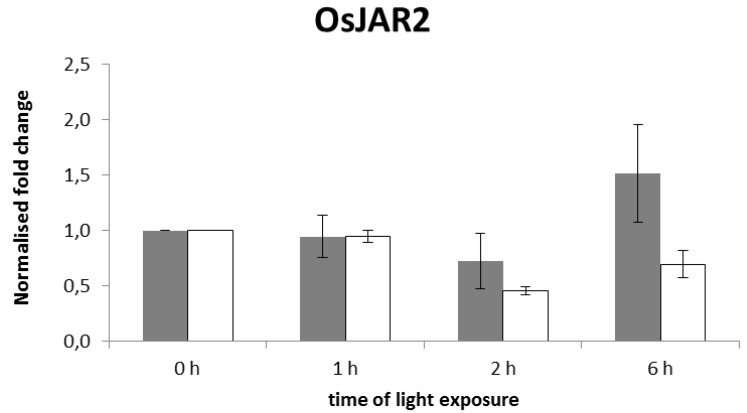
Transcript levels of *OsJAR2* in wild type and mutant seedlings in response to blue light treatment. Nipponbare (grey bar) and *phyAphyC* mutant (white bar) seedlings were irradiated with blue light (10 µmol/m^2^s) for the times indicated. qPCR analysis was carried out using two standard genes, eEF-1α and OsUBI5, for normalisation. Data represent the average of three independent experiments. No statistically significant induction of *OsJAR2* was found.

In addition to this unknown JA-dependent branching point responsible for blue-light dependent growth control, there seems to be a branching of the biosynthetic pathway leading to JA-Ile. The higher production of JA-Ile in the phyAphyC mutant contrasts with the severely impaired induction of OsJAR1 transcripts. It might, therefore, be possible that a second enzyme, different from OsJAR1 contributes to the biosynthesis of JA-Ile in young rice seedlings. This assumption would be consistent with our previous observation that JA-Ile still can be synthesized in *osjar1* mutants [31]. It seems to be difficult to identify this enzyme via its transcriptional regulation. At least we found that a putative candidate, *OsJAR2* [31,41], is not regulated on the transcriptional level (Figure A1).

Comparing the temporal regulation of JA levels and gene-expression, we found that *OsJAZ8*, which is not fully induced in *ojsar1* mutants [31], does not respond stronger in *phyAphyC* mutants, although there is a higher amount of active JA present. This finding either means that the induction of *OsJAZ8* transcripts is already saturated by the lower JA levels present in the wild type, or that the expression of this specific *JAZ* gene in blue light is not dependent on phytochrome, or alternatively, that factors different from Jasmonates contribute to the regulation of the gene.

### 2.4. Less Jasmonates Are Produced in *phyAphyC* in Response to Mechanical Wounding

The results of gene expression analysis and phytohormone levels in *phyAphyC* seedlings raised the question whether the high induction of JA and JA-Ile after blue light might be limited to seedling stage and light treatment or whether other treatments would induce such high JA-amounts as well. We therefore applied a different treatment (wounding), which is known to induce the biosynthesis of JA, at a different developmental stage. In order to wound plants in a reproducible manner, we used the MecWorm system, which mimics the mechanical part of insect feeding [31,42].

**Figure 4:**
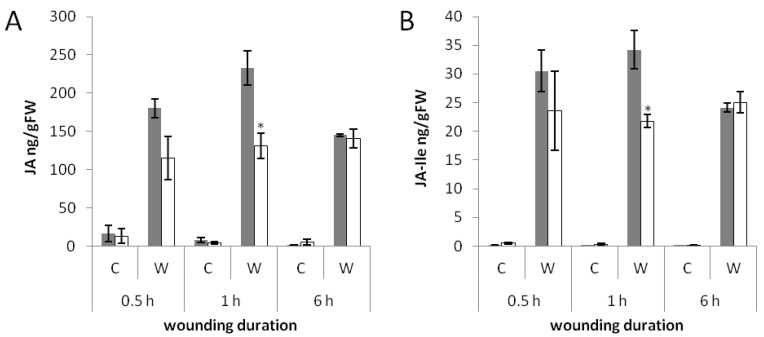
JA (**A**) and JA-Ile (**B**) levels of rice plants in response to wounding. Plants of wild type (grey bar) and *phyAphyC* (white bar) were wounded with MecWorm six weeks after germination for the indicated time intervals. Hormone levels are depicted relative to sample fresh weight and represented as an average of three independent experiments. C = control plant, W = wounded plant. Statistically significant differences to the wild type are indicated by an asterisk (Student’s *t*-test, *p* < 0.05).

We treated rice plants six weeks after germination for specific time intervals using the MecWorm system and measured the hormone levels. In both wild type and *phyAphyC* mutant, JA and JA-Ile were induced due to the wounding treatment (Figure 4A,B). JA and JA-Ile were already strongly induced after 30 min of wounding (wild type: 180.3 ng/gFW and 30.5 ng/gFW, respectively, mutant: 115.2 ng/gFW and 23.6 ng/gFW, respectively). After 1 h of continuous wounding, wild type leaves contained twice the content of JA compared to *phyAphyC* (wild type: 232.8 ng/gFW, mutant: 131.1 ng/gFW; Figure 4A). At the same time point, *phyAphyC* also produced significantly less JA-Ile than the wild type (wild type: 34.2 ng/gFW, mutant: 21.7 ng/gFW; Figure 4B). However, after 6 h of treatment with MecWorm, both Nipponbare and *phyAphyC* showed similar hormone levels, because the wild type response had ceased somewhat decreasing to the levels in *phyAphyC*. In summary, wild type leaves developed a transient peak of JA and JA-Ile after 1 h of wounding, whereas in *phyAphyC*, both hormones remained at the level reached after 30 min.

Thus, in contrast to the blue-light response of seedlings, in MecWorm-treated leaves the levels of JA or JA-Ile were not increased in the *phyAphyC* mutant compared to the wild type. We even found that *phyAphyC* produces less JA and JA-Ile than the wild type, when measured after 1 h of MecWorm treatment. Hence, the biosynthesis of JA and JA-Ile is not generally enhanced in *phyAphyC* mutants, but depending on defined developmental stages and stimuli. 

### 2.5. Transcriptional Regulation of JA-Responsive Genes by Continuous Mechanical Wounding

In response to blue light, the induction of *OsJAR1* was impaired in *phyAphyC* while two further JA-responsive genes, *OsAOC* and *OsJAZ8*, were induced as in the wild type (Figure 2). Therefore, we examined the expression of the same genes after wounding with MecWorm. 

**Figure 5:**
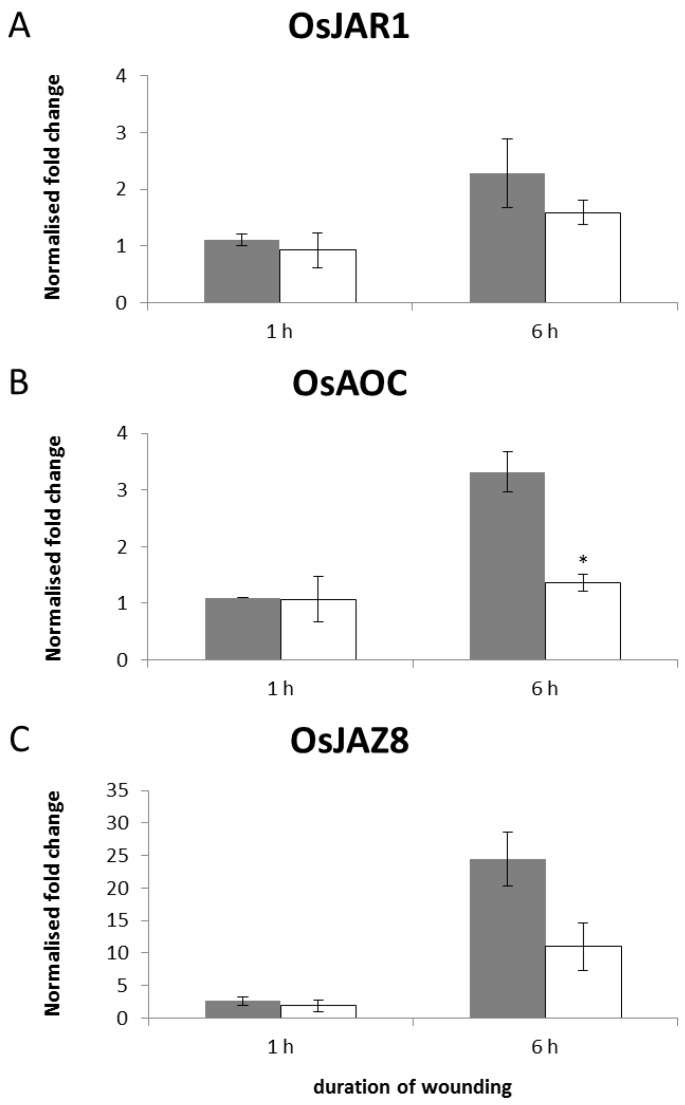
Transcript levels of the representative JA-biosynthesis *OsJAR1* (**A**) and *OsAOC* (**B**), and the JA-signaling gene *OsJAZ8* (**C**) in wild type and mutant plants in response to wounding. Plants of wild type (grey bar) and *phyAphyC* (white bar) were wounded with MecWorm six weeks after germination for the indicated time intervals. qPCR analysis was carried out using two standard genes, eEF-1α and OsUBI5, for normalization. The fold change induction for each gene was calculated relative to a corresponding untreated control plant. Data represent the average of three independent experiments. Statistically significant differences to the wild type are indicated by an asterisk (Student’s *t*-test, *p* < 0.05).

After 1 h of MecWorm-treatment, two of the genes were not induced at all (*OsJAR1*, *OsAOC*) and the third gene (*OsJAZ8*) was only induced weakly (Figure 5). None of these genes revealed a difference between wild type and *phyAphyC*.

After 6 h of treatment with MecWorm, transcription of *OsAOC* was induced three-fold and *OsJAZ8* 25-fold in the wild type (Figure 5B,C), relative to a non-treated control. The *OsJAR1* transcript was induced only slightly (approximately two-fold; Figure 5A).

In the mutant, the expression of *OsJAR1* in leaves treated for 6 h was only weakly induced with 1.8-fold induction relative to the control, comparable to the results obtained from the wild type (Figure 5A).

The gene expression of *OsAOC*, however, seemed to be affected by the *phyAphyC* mutation. In the mutant, no induction of *OsAOC* could be detected at 6 h treatment (Figure 5B) while in the wild type *OsAOC* was three-fold induced relative to a control plant. The expression of *OsJAZ8* after 6 h MecWorm treatment was reduced in *phyAphyC* by a factor of 2 as compared to the wild type (24.4-fold in the wild type *vs*. 11.0-fold in the mutant, Figure 5C) but the statistical significance of this difference was above the confidence level of 0.05 in a Student’s *t*-test.

Despite the impaired *OsAOC*-induction in *phyAphyC*, the JA-level was strongly elevated in the mutant as compared to the control (Figure 4A). This suggests that transcriptional activation of *OsAOC* is not required to sustain the induction of JA in response to wounding.

Compared to the gene expression of blue light irradiated seedlings, the regulation of JA-dependent genes differs in the MecWorm-wounded rice plants. While in the seedlings only *OsJAR1* was expressed differentially in wild type and mutant, in the adult wounded plants it is *OsAOC*. 

Whether a given gene of the jasmonate pathway is sensitive to the control by photolabile phytochrome or not, is, thus, not a constant feature of jasmonate signaling, but depends on developmental state and on the quality of stimulation. Trewavas [43] has worked out, that the developmental responses to phytohormones are controlled by the responsiveness of the target tissue rather than by the abundance of the signal. The finding that the phy-dependent changes of jasmonate status depend on developmental state and signal context, *i.e.*, on the competence of the target cells, provides a neat illustration for this point. 

## 3. Experimental

### 3.1. Plant Material

The *phyAphyC* double mutant (*phyA-2phyC-1* alleles) line was generated by crossing of *phyA* and *phyC* single mutants, which had been obtained by Tos17-mediated mutagenesis. All these mutant lines are described in detail by Takano *et al*. [10]. 

The wild type background for the *phyAphyC* line is *Oryza sativa* L. ssp. *japonica* cv. Nipponbare, which was used as a control in this study.

All seeds used for the experiments were propagated in the greenhouses of the Botanical Garden of the KIT (Karlsruhe Institute of Technology, Karlsruhe, Germany).

### 3.2. Plant Cultivation and Treatments

Rice seeds were dehusked and surface sterilized before sowing. Seeds were shaken in 70% ethanol for 1 min and washed with ultrapure H_2_O two times. Afterwards, the seeds were incubated in a solution of sodium-hypochlorite (5% w/v, Carl Roth, Karlsruhe, Germany), shaking at 100 rpm for 20 min, and then washed with sterile ultrapure H_2_O under sterile conditions.

Subsequently, 20 to 25 seeds were sown into one magenta box (Sigma-Aldrich, St. Louis, MO, USA) on phytoagar (0.6% w/v, Duchefa, Haarlem, The Netherlands). 

Seedlings were irradiated with blue light (λ_max_ 470 nm), red light (λ_max_ 650 nm), or far-red light (λ_max_ 735 nm) using custom made LED arrays as described by Qiao *et al*. [44] adjusting to identical fluence rates (10 µmol/m^2^s).

For MecWorm treatments, seeds were sown onto 0.4% phytoagar and raised at 25 °C under continuous white light (20 µmol/m^2^s). After two weeks, seedlings were transferred to sand and raised for four additional weeks in a phytochamber under short-day conditions (10 h light at 28 °C, 280 µmol/m^2^s, 14 h darkness at 22 °C). Once a week plants were fertilized (Wuxal “TopN” and “Super” fertilizers, Manna, Ammerbuch-Pfäffingen, Germany).

The wounding experiments were conducted using the MecWorm system [42]. The youngest, fully developed leaf of a rice plant raised for six weeks was treated for variable time intervals. As a control, the respective leaf of a non-treated plant of the same age, raised in the same phytochamber, was harvested. The details for the MecWorm treatments have been described previously in Svyatyna *et al*. [31].

### 3.3. Hormone Analysis

For light experiments, plant material was harvested immediately after irradiation and transferred to liquid nitrogen. Fresh weight of each frozen sample was recorded very exactly using an analytical balance, and subsequently the plant material was freeze-dried. Plant hormone extraction and analysis was carried out as described in Svyatyna *et al*. [31].

After wounding experiments, treated and control leaves were harvested directly and frozen in liquid nitrogen. Frozen sample material was ground roughly and fresh weight was measured using an analytical balance. Samples were stored at −80 °C until shipping on dry ice for further analysis. Hormones were extracted by methanol and extracts analyzed using a Zorbax Eclipse XDB-C18 column (50 × 4.6 mm, 1.8 µm, Agilent Technologies, Waldbronn, Germany) installed on an Agilent 1200 HPLC system (Agilent Technologies, Böblingen, Germany) coupled to an API 5000 tandem mass spectrometer (Applied Biosystems, Darmstadt, Germany) as described in Svyatyna *et al*. [31].

### 3.4. Gene Expression Analysis

Plant material was harvested directly after the end of the respective treatment and frozen in liquid nitrogen. Sample material was stored at −80 °C until RNA extraction.

Samples were homogenized into a frozen powder (TissueLyser, Qiagen, Hilden, Germany) and then RNA was extracted using the innuPREP Plant RNA Kit (Analytik Jena, Jena, Germany) according to the manufacturer’s instructions, including the steps to remove genomic gDNA contamination by RNAse-free DNAse (Qiagen, Hilden, Germany). 

Only RNA with high quality and integrity, as verified via spectrophotometry and gel electrophoresis, was used for cDNA-synthesis with the DyNAmo cDNA-Sythesis Kit (Thermo Fisher Scientific Inc., Waltham, MA, USA) using oligo-dT primers.

The qPCR analysis was performed using protocol and primers already described in Svyatyna *et al*. [31]. This also applies to the calculation of relative gene expression, which was carried out using the ΔΔCt method, with eEF-1α and OsUBI5 as internal control genes for normalization.

### 3.5. Statistical Analysis

Data obtained in this study were examined with two different statistical methods, Student’s *t*-test and Tukey-Kramer test, with the same results. Confidence levels of Student’s *t*-tests were depicted in the respective graphs, one asterisk indicating *p* < 0.05, two asterisks indicating *p* < 0.01.

## 4. Conclusions

**Figure A2:**
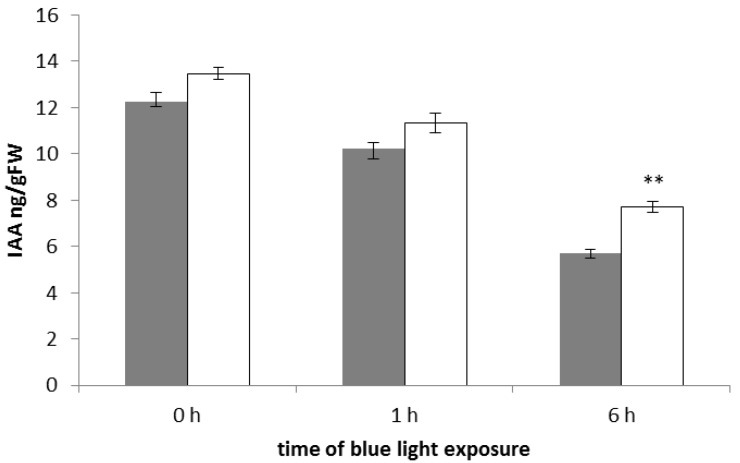
IAA levels of rice seedlings in response to blue light. Etiolated wild type (grey bars) and *phyAphyC* (white bars) seedlings were irradiated with blue light (10 µmol/m^2^s) at day 5 for the times indicated. Hormone levels are shown relative to sample fresh weight and represent an average of three independent experiments. Statistically significant differences to the wild type are indicated by two asterisks (Student’s *t*-test, *p* < 0.01).

Based on previous findings, that the response to light in rice seedlings depends on jasmonate, and that the transcriptional activation of OsJAR1 is impaired in the *phyAphyC* mutant, we examined the transcriptional and hormonal regulation in this mutant. Surprisingly, JA and JA-Ile were produced at higher levels in *phyAphyC*, and two JA-dependent genes investigated in this study were transcriptionally activated by blue light in the same way as in the wild type. We conclude that the effect of blue light on the activation of JA and JA-Ile biosynthesis proceeds independently of photolabile phytochrome through different blue-light sensitive receptors. Interestingly, although both JA and JA-Ile are present at higher levels after extended exposure to blue light, coleoptiles of *phyAphyC* mutants elongate more compared to the wild type when raised in continuous blue light. Hence, the light response of coleoptile elongation could be mediated by JA derivatives different from JA and JA-Ile. Alternatively, the increased levels of jasmonates in the mutants might affect other hormone pathways such as auxin. The negative regulation of JA biosynthesis by photolabile phytochrome is specific for the light response of coleoptile elongation, and not observed for the wound response of leaves. We conclude that phys in concert with other blue light sensitive receptors regulate the JA-dependent responses to blue light in rice seedlings. Phys might control important enzymatic steps in the metabolism and activation of JA, independent of the canonical positive feedback mechanism of JA on the genes of its own biosynthesis pathway. This model is supported by our observation that the transcription of *OsJAR1* is not induced in *phyAphyC* although this mutant accumulates even more JA than the wild type. Furthermore, we observed increased auxin levels in the *phyAphyC* mutant, in both etiolated and deetiolated seedlings. It is possible that the higher amount of auxin in mutant coleoptiles (Figure A2) contribute to enhanced coleoptile elongation. 

**Figure 6:**
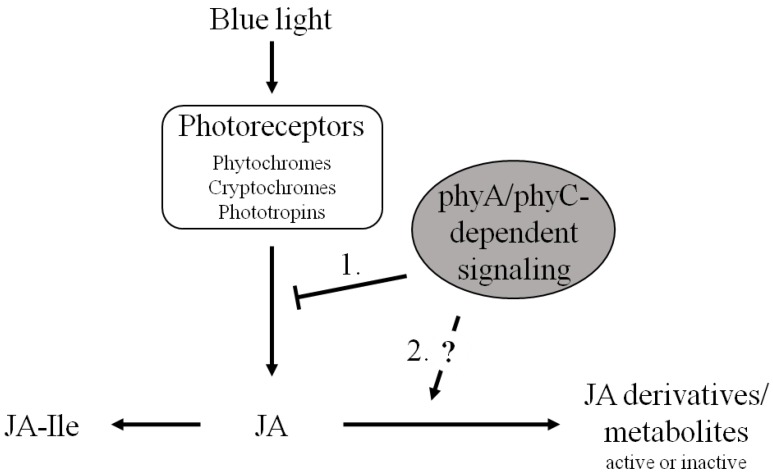
Working model for the modulation of JA- biosynthesis and -metabolism by phyA and phyC during the blue-light response in rice seedlings. Blue light is perceived by photoreceptors (including phy and other blue-light sensitive receptors) leading to initial induction of JA-biosynthesis. PhyA/phyC-dependent signaling constrains JA-synthesis below a certain threshold by either inhibiting further JA-biosynthesis (1) or by inducing JA-depletion via formation of other JA-derivatives apart from JA-Ile (2).

Based on our results, we developed a working hypothesis for the role of phyA and phyC in the blue-light induced JA-response. The biosynthesis of JA is induced by blue light, mediated via photoreceptors (including phys, but also other blue-light sensitive receptors). Due to the autocatalytic regulation of JA-synthesis, this would result in a dramatic overshoot of the JA pathway (as characteristic for the wound response). In case of the light response, an equilibrium is formed by a negative feedback, which is, at least partially, mediated by phyA/phyC-dependent signaling via an unknown mechanism (Figure 6). When these photoreceptors are impaired, this negative feedback is removed leading to a severe JA-overproduction. Whether this phy-dependent JA-homeostasis during photomorphogenesis is achieved by inhibition of JA-biosynthesis or by scavenging of JA via induction of synthesis of further JA-derivatives, remains to be elucidated. In order to examine, whether the activity of specific enzymes metabolizing JA to active derivatives is indeed controlled by phyA and phyC we will extend the current investigation to additional photoreceptor mutants, such as cry and phot, as well as combinations of different photoreceptor mutations.

## References

[1] Briggs W.R., Christie J.M. (2002). Phototropins 1 and 2: Versatile plant blue-light receptors. Trends Plant Sci..

[2] Christie J.M. (2007). Phototropin blue-light receptors. Annu. Rev. Plant Biol..

[3] Lin C., Shalitin D. (2003). Cryptochrome structure and signal transduction. Annu. Rev. Plant Biol..

[4] Sancar A. (2003). Structure and function of DNA photolyase and cryptochrome blue-light photoreceptors. Chem. Rev..

[5] Furuya M. (1993). Phytochromes—Their Molecular Species, Gene Families, and Functions. Annu. Rev. Plant Physiol. Plant Mol. Biol..

[6] Nagy F., Schäfer E. (2002). Phytochromes control photomorphogenesis by differentially regulated, interacting signaling pathways in higher plants. Annu. Rev. Plant Biol..

[7] Quail P.H. (1991). Phytochrome: A light-activated molecular switch that regulates plant gene expression. Annu. Rev. Genet..

[8] Smith H. (1995). Physiological and Ecological Function within the Phytochrome Family. Annu. Rev. Plant Physiol. Plant Mol. Biol..

[9] Takano M., Inagaki N., Xie X., Kiyota S., Baba-Kasai A., Tanabata T., Shinomura T. (2009). Phytochromes are the sole photoreceptors for perceiving red/far-red light in rice. Proc. Natl. Acad. Sci. USA.

[10] Takano M., Inagaki N., Xie X., Yuzurihara N., Hihara F., Ishizuka T., Yano M., Nishimura M., Miyao A., Hirochika H. (2005). Distinct and cooperative functions of phytochromes A, B, and C in the control of deetiolation and flowering in rice. Plant Cell.

[11] Smith H., Holmes M.G. (1977). The function of phytochrome in natural-environment—III. Measurement and calculation of phytochrome photo-equilibria. Photochem. Photobiol..

[12] Botto J.F., Sanchez R.A., Whitelam G.C., Casal J.J. (1996). Phytochrome a mediates the promotion of seed germination by very low fluences of light and canopy shade light in *Arabidopsis*. Plant Physiol..

[13] Pjon C.-J., Furuya M. (1967). Phytochrome action in *Oryza sativa* L.: I. Growth responses of etiolated coleoptiles to red, far-red and blue light. Plant Cell Physiol..

[14] Xie X., Shinomura T., Inagaki N., Kiyota S., Takano M. (2007). Phytochrome-mediated inhibition of coleoptile growth in rice: Age-dependency and action spectra. Photochem. Photobiol..

[15] Hirose F., Shinomura T., Tanabata T., Shimada H., Takano M. (2006). Involvement of rice cryptochromes in de-etiolation responses and flowering. Plant Cell Physiol..

[16] Fujioka S., Li J., Choi Y.H., Seto H., Takatsuto S., Noguchi T., Watanabe T., Kuriyama H., Yokota T., Chory J. (1997). The Arabidopsis *deetiolated2* mutant is blocked early in brassinosteroid biosynthesis. Plant Cell.

[17] Li J., Nagpal P., Vitart V., McMorris T.C., Chory J. (1996). A role for brassinosteroids in light-dependent development of Arabidopsis. Science.

[18] Haga K., Iino M. (2004). Phytochrome-mediated transcriptional up-regulation of ALLENE OXIDE SYNTHASE in rice seedlings. Plant Cell Physiol..

[19] Riemann M., Müller A., Korte A., Furuya M., Weiler E.W., Nick P. (2003). Impaired induction of the jasmonate pathway in the rice mutant hebiba. Plant Physiol..

[20] Svyatyna K., Riemann M. (2012). Light-dependent regulation of the jasmonate pathway. Protoplasma.

[21] Wasternack C., Hause B. (2013). Jasmonates: Biosynthesis, perception, signal transduction and action in plant stress response, growth and development. An update to the 2007 review in Annals of Botany. Ann. Bot..

[22] Staswick P.E., Tiryaki I. (2004). The oxylipin signal jasmonic acid is activated by an enzyme that conjugates it to isoleucine in Arabidopsis. Plant Cell.

[23] Sheard L.B., Tan X., Mao H., Withers J., Ben-Nissan G., Hinds T.R., Kobayashi Y., Hsu F.F., Sharon M., Browse J. (2010). Jasmonate perception by inositol-phosphate-potentiated COI1-JAZ co-receptor. Nature.

[24] Xie D.X., Feys B.F., James S., Nieto-Rostro M., Turner J.G. (1998). COI1: An *Arabidopsis* gene required for jasmonate-regulated defense and fertility. Science.

[25] Chini A., Fonseca S., Fernandez G., Adie B., Chico J.M., Lorenzo O., Garcia-Casado G., Lopez-Vidriero I., Lozano F.M., Ponce M.R. (2007). The JAZ family of repressors is the missing link in jasmonate signalling. Nature.

[26] Thines B., Katsir L., Melotto M., Niu Y., Mandaokar A., Liu G., Nomura K., He S.Y., Howe G.A., Browse J. (2007). JAZ repressor proteins are targets of the SCF(COI1) complex during jasmonate signalling. Nature.

[27] Riemann M., Haga K., Shimizu T., Okada K., Ando S., Mochizuki S., Nishizawa Y., Yamanouchi U., Nick P., Yano M. (2013). Identification of rice Allene Oxide Cyclase mutants and the function of jasmonate for defence against *Magnaporthe oryzae*. Plant. J..

[28] Riemann M., Bouyer D., Hisada A., Müller A., Yatou O., Weiler E.W., Takano M., Furuya M., Nick P. (2009). Phytochrome A requires jasmonate for photodestruction. Planta.

[29] Sineshchekov V.A., Loskovich A.V., Riemann M., Nick P. (2004). The jasmonate-free rice mutant *hebiba* is affected in the response of phyA'/phyA" pools and protochlorophyllide biosynthesis to far-red light. Photochem. Photobiol. Sci..

[30] Radhika V., Kost C., Mithöfer A., Boland W. (2010). Regulation of extrafloral nectar secretion by jasmonates in lima bean is light dependent. Proc. Natl. Acad. Sci. USA.

[31] Svyatyna K., Jikumaru Y., Brendel R., Reichelt M., Mithöfer A., Takano M., Kamiya Y., Nick P., Riemann M. (2013). Light induces jasmonate-isoleucine conjugation via OsJAR1-dependent and -independent pathways in rice. Plant Cell Environ..

[32] Riemann M., Takano M. (2008). Rice JASMONATE RESISTANT 1 is involved in phytochrome and jasmonate signalling. Plant Cell Environ..

[33] Yamada S., Kano A., Tamaoki D., Miyamoto A., Shishido H., Miyoshi S., Taniguchi S., Akimitsu K., Gomi K. (2012). Involvement of OsJAZ8 in jasmonate-induced resistance to bacterial blight in rice. Plant Cell Physiol..

[34] Terol J., Domingo C., Talon M. (2006). The GH3 family in plants: Genome wide analysis in rice and evolutionary history based on EST analysis. Gene.

[35] Jain M., Kaur N., Tyagi A.K., Khurana J.P. (2006). The auxin-responsive GH3 gene family in rice (*Oryza sativa*). Funct. Integr. Genomics.

[36] Ye H., Du H., Tang N., Li X., Xiong L. (2009). Identification and expression profiling analysis of TIFY family genes involved in stress and phytohormone responses in rice. Plant Mol. Biol..

[37] Costigan S.E., Warnasooriya S.N., Humphries B.A., Montgomery B.L. (2011). Root-localized phytochrome chromophore synthesis is required for photoregulation of root elongation and impacts root sensitivity to jasmonic acid in *Arabidopsis*. Plant Physiol..

[38] Zhai Q., Li C.B., Zheng W., Wu X., Zhao J., Zhou G., Jiang H., Sun J., Lou Y., Li C. (2007). Phytochrome chromophore deficiency leads to overproduction of jasmonic acid and elevated expression of jasmonate-responsive genes in *Arabidopsis*. Plant Cell Physiol..

[39] Chen J., Sonobe K., Ogawa N., Masuda S., Nagatani A., Kobayashi Y., Ohta H. (2013). Inhibition of arabidopsis hypocotyl elongation by jasmonates is enhanced under red light in phytochrome B dependent manner. J. Plant Res..

[40] Sun J., Xu Y., Ye S., Jiang H., Chen Q., Liu F., Zhou W., Chen R., Li X., Tietz O. (2009). *Arabidopsis* ASA1 is important for jasmonate-mediated regulation of auxin biosynthesis and transport during lateral root formation. Plant Cell.

[41] Wakuta S., Suzuki E., Saburi W., Matsuura H., Nabeta K., Imai R., Matsui H. (2011). OsJAR1 and OsJAR2 are jasmonyl-l-isoleucine synthases involved in wound- and pathogen-induced jasmonic acid signalling. Biochem. Biophys. Res. Commun..

[42] Mithöfer A., Wanner G., Boland W. (2005). Effects of feeding *Spodoptera littoralis* on lima bean leaves. II. Continuous mechanical wounding resembling insect feeding is sufficient to elicit herbivory-related volatile emission. Plant Physiol..

[43] Trewavas A.J. (1982). Growth substance sensitivity—The limiting factor in plant development. Physiol. Plant..

[44] Qiao F., Petrášek J., Nick P. (2010). Light can rescue auxin-dependent synchrony of cell division in a tobacco cell line. J. Exp. Bot..

